# A Lymphoma Almost Overlooked

**DOI:** 10.7759/cureus.2110

**Published:** 2018-01-24

**Authors:** Jeremy Lorber, Martina Zalom

**Affiliations:** 1 Hematology and Oncology, Olive View Ucla

**Keywords:** diffuse large b-cell lymphoma, dlbcl, aids, hiv, vaginal cancer, synchronous malignancies

## Abstract

A 50-year-old female with a prolonged history of untreated human immunodeficiency virus (HIV) presented with a large vaginal mass. During workup, the mass was found to be vaginal squamous cell carcinoma. Imaging suggested stage IV disease, but a biopsy of liver lesions demonstrated synchronous diffuse large B-cell lymphoma. Her treatment course was notable for complete remission of her lymphoma with lymphoma-directed chemotherapy and complete clinical response of her squamous cell carcinoma to lymphoma-directed therapy. She tolerated intensive chemotherapy despite her HIV but eventually died due to infectious complications during surgery to address a vaginal fistula. The case is demonstrative of several important diagnostic and therapeutic principles in the management of HIV-associated malignancies. Thorough consideration and testing must be performed to ensure accurate staging, as synchronous malignancies and infections can distort standard clinical testing. Further, standard chemotherapeutic regimens often must be tailored and specially sequenced when dealing with severely immunocompromised patients with multiple synchronous processes.

## Introduction

Human immunodeficiency virus (HIV) is well known to be associated with various malignancies. The workup and treatment of HIV-associated malignancy is fraught with unique pitfalls due to concomminent infections which may mimic malignancy, synchronous malignancies which may be overlooked and attributed to the primary diagnosis, and complications of treatment due to immunosuppression. The following case demonstrates these unique challenges during diagnosis, treatment selection, treatment monitoring, and managment of complications.

## Case presentation

**Figure 1:**
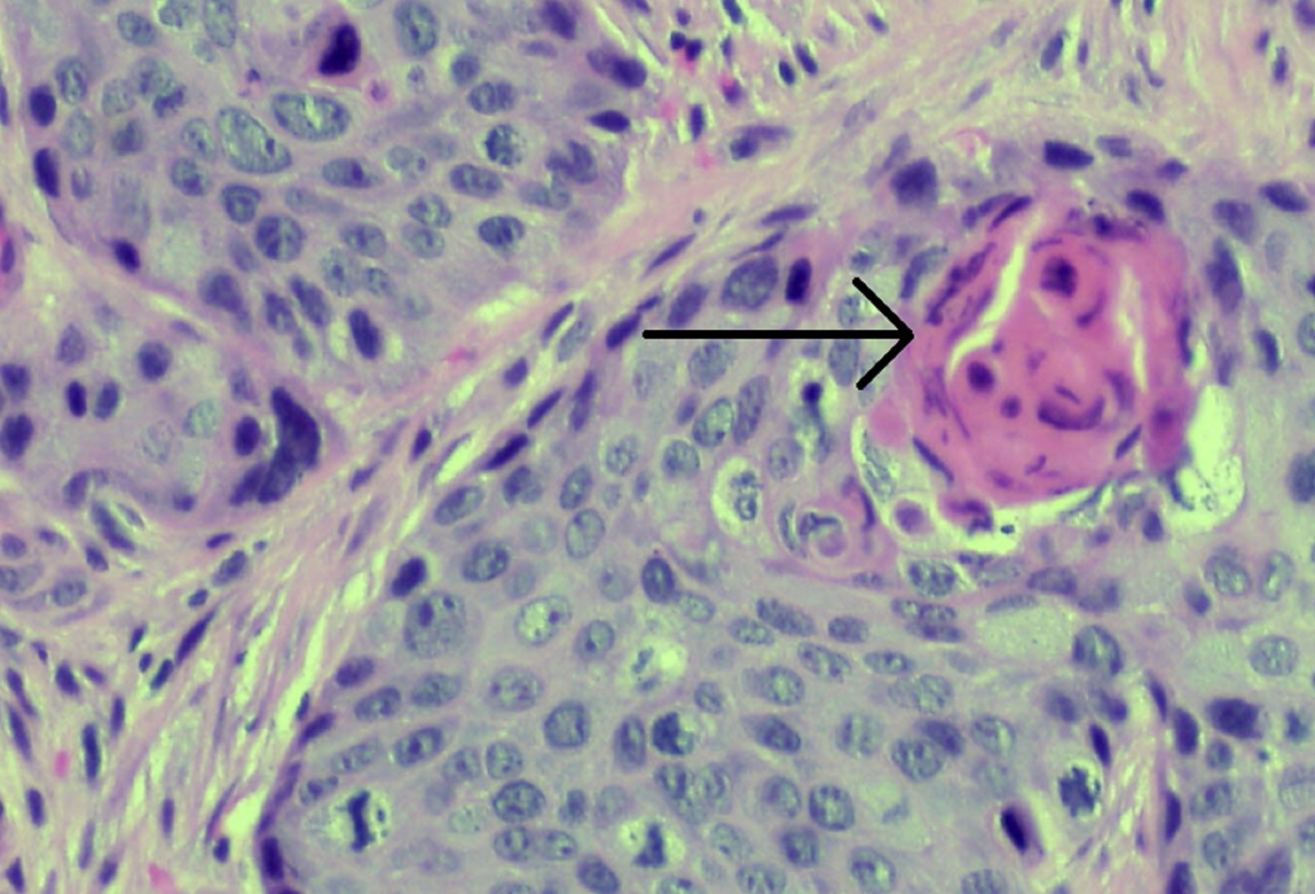
Vaginal squamous cell carcinoma with keratin pearl

**Figure 2:**
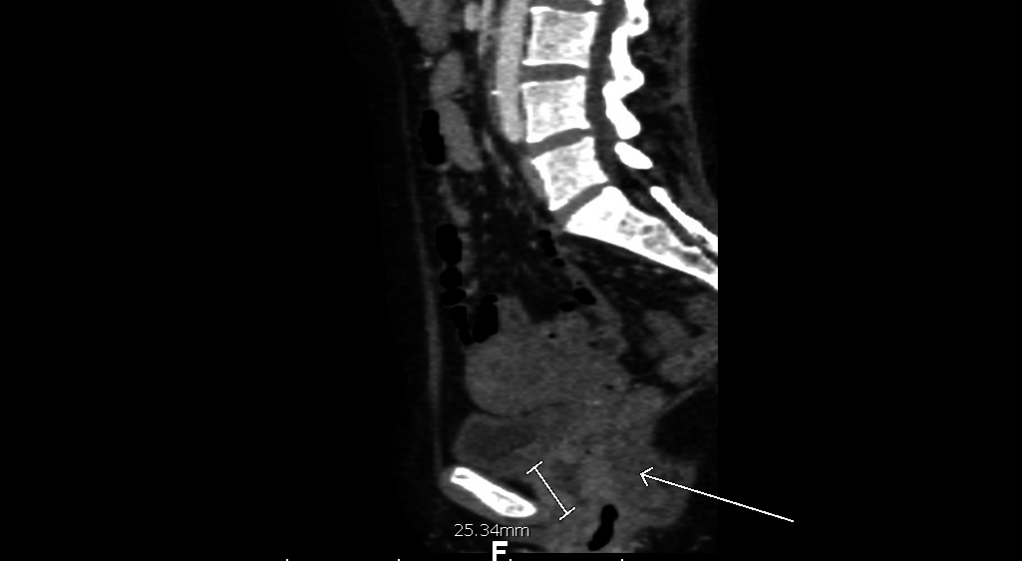
Infiltrating pelvic mass on computerized tomography

**Figure 3:**
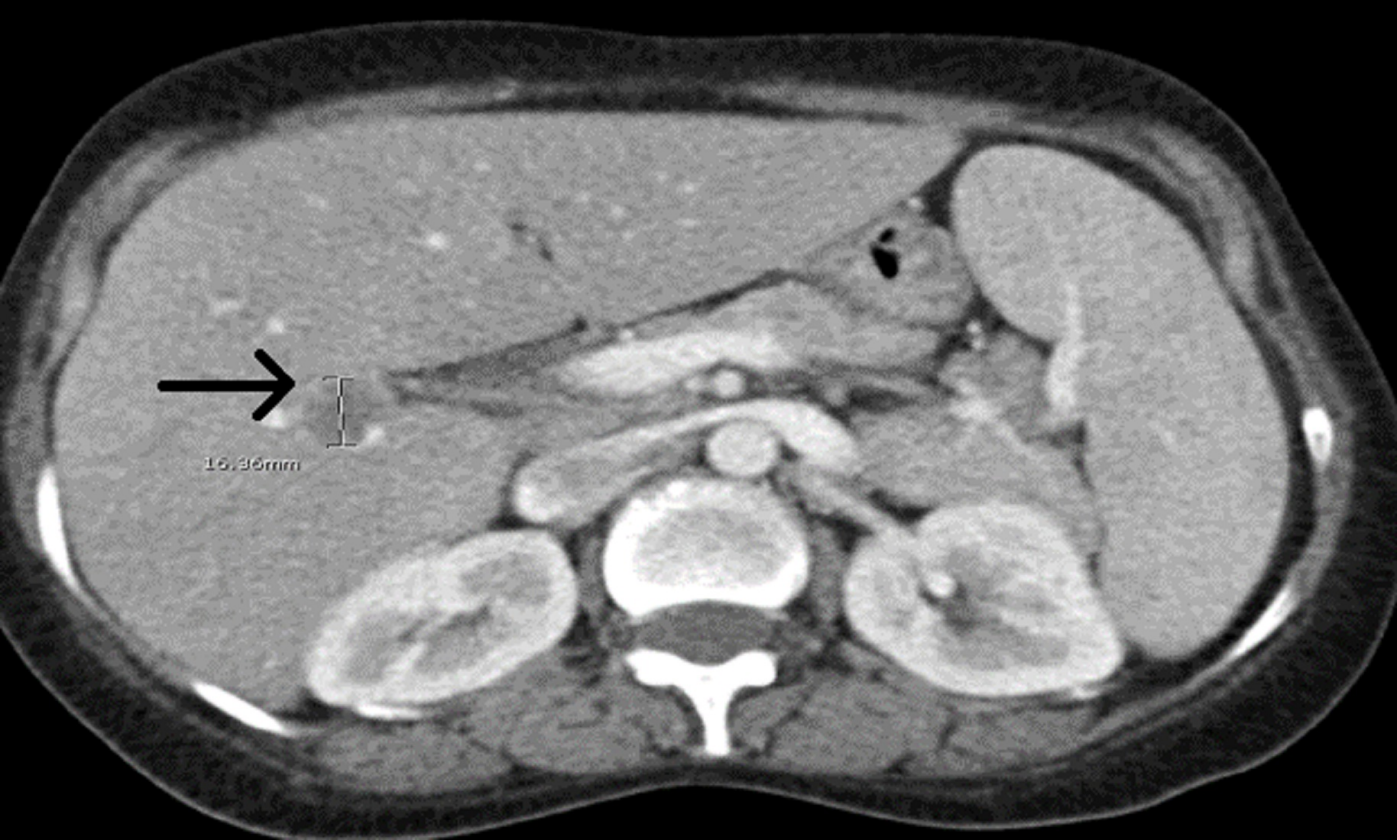
Hepatic mass seen on computerized tomography, initially presumed to be metastatic vaginal cancer

**Figure 4:**
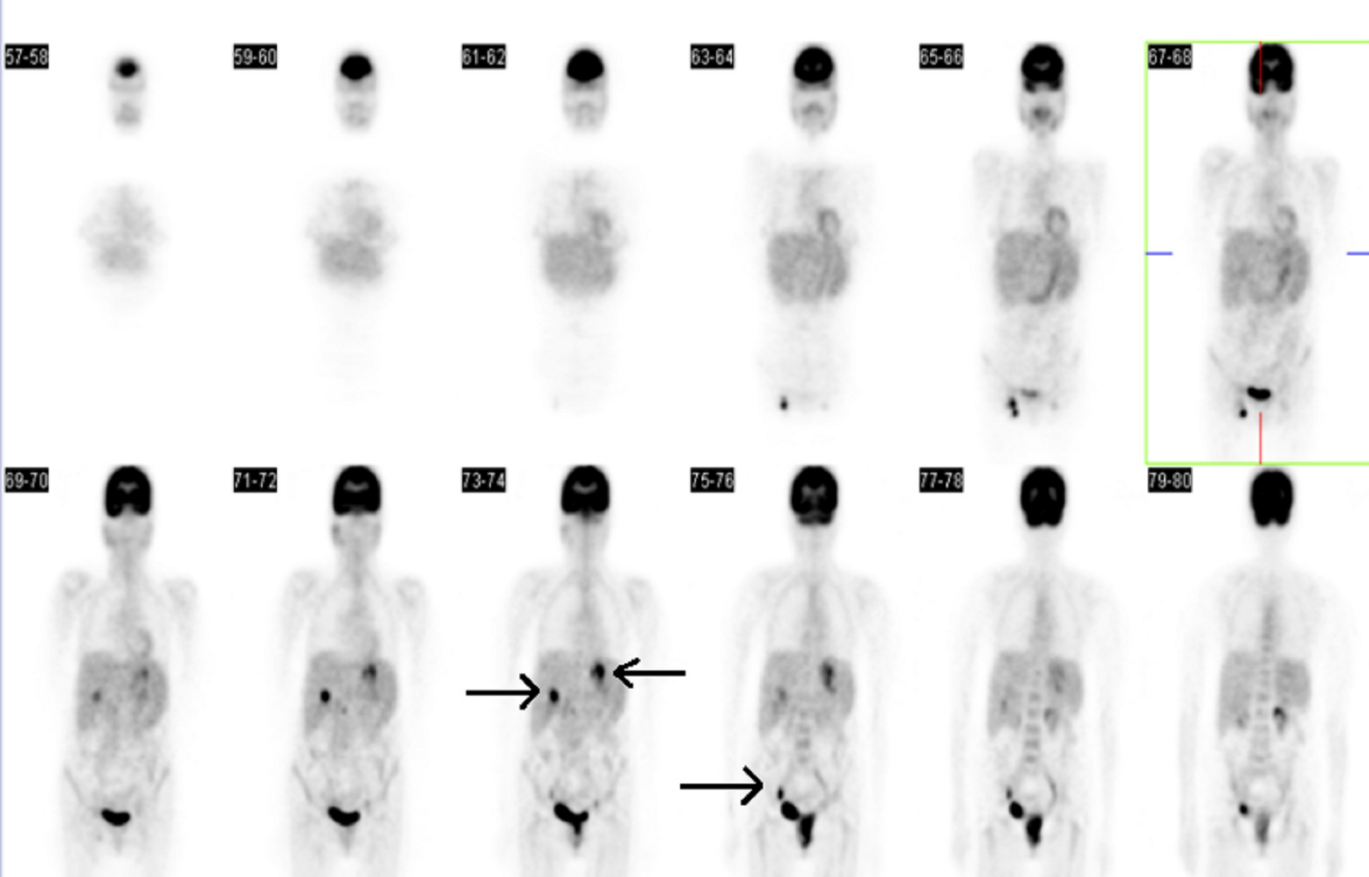
Positron-emission tomography with hypermetabolic activity in pelvis and various pelvic and abdominal nodal regions

A 50-year-old female with an 18-year history of untreated HIV infection presented with vaginal bleeding and discharge for one month. She also endorsed an unintentional 10-pound weight loss over the same period. On exam, her vital signs were normal. She appeared thin but in no distress. Her genitourinary exam revealed a 6 cm in diameter fungating vaginal tumor invading the anterior wall with evidence of bladder invasion later discovered on cystoscopy. The exam also revealed bilateral inguinal lymphadenopathy. Initial laboratory studies were remarkable for a white blood cell count of 4.3 x 10^9^/L, hemoglobin 10.4 g/dL, and platelets 240 × 10^9^/L. The CD4 lymphocyte count was 164 cells/mm^3^. Given the concern for primary gynecological malignancy, a biopsy was performed on the vaginal mass, with pathology consistent with vaginal squamous cell carcinoma (Figure 1). Staging computed tomography (CT) showed a 2.5 cm mass infiltrating the fat planes between the vaginal wall and bladder, prominent lymph nodes in the gastrohepatic space, porta hepatis, portocaval stations, retroperitoneum, internal and external iliac, and inguinal stations, and a solid low density mass in the right lobe of the liver concerning for metastatic disease (Figures 2-3). Positron-emission tomography (PET) revealed an intensely hypermetabolic vaginal mass, hypermetabolic lymph nodes within the splenic hilum, portocaval, portahepatic, and bilateral external iliac and bilateral inguinal regions, and an intensely hypermetabolic lesion in the middle right hepatic lobe, which was suspicious for hepatic metastasis (Figure 4).

**Figure 5:**
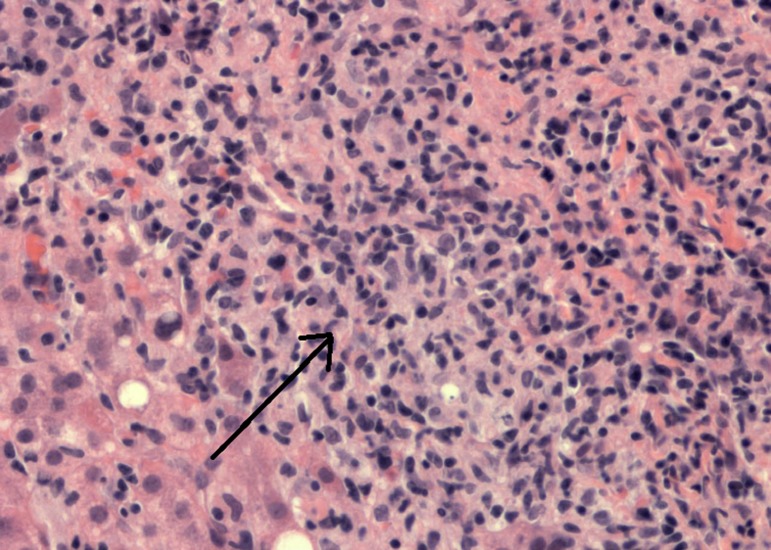
Liver biopsy with diffuse large B-cell lymphoma

The patient was initially scheduled for systemic chemotherapy for diagnosis of stage IV vaginal squamous cell carcinoma. Due to her prolonged history of untreated HIV and concern that the hypermetabolic hepatic lesion may represent a process other than metastatic vaginal carcinoma, the patient underwent hepatic core needle biopsy. Pathology showed a population of large atypical lymphocytes positive for CD20, BCL2, BCL6, and negative for CD10 (Figure 5). Extensive necrosis and scant sample made impossible determination of MUM1 staining or determination of cMYC expression and translocation. The additional diagnosis of diffuse large B-cell lymphoma (DLBLC) was given.

**Figure 6:**
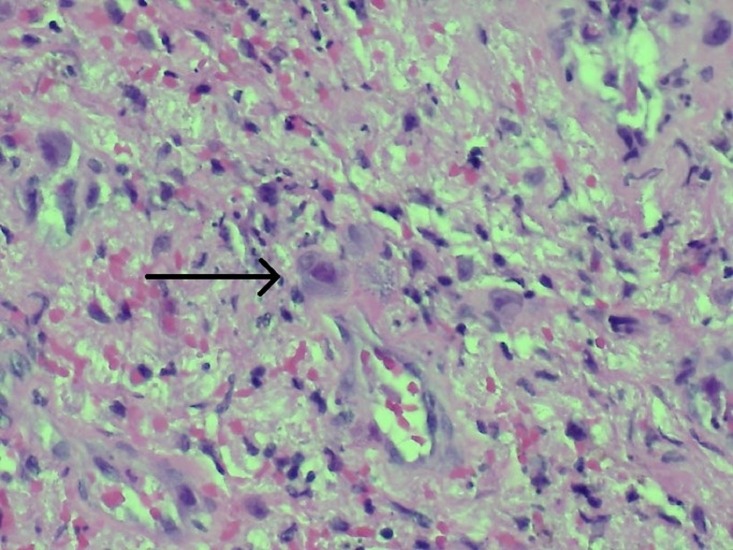
Biopsy of hypermetabolic inguinal node with cytomegalovirus, no lymphoma

The treatment of the patient’s vaginal carcinoma was deferred because of the new concomitant diagnosis of a high-grade lymphoma. The patient was administered a chemotherapy regimen consisting of rituximab, etoposide, doxorubicin, vincristine, cyclophosphamide, and dexamethasone (R-EPOCH) and initiated on HIV therapy. After the second cycle of R-EPOCH, PET revealed persistent inguinal node activity, concerning for refractory disease. Core needle biopsy of the inguinal node, however, revealed cytomegalovirus (CMV) adenitis without evidence of lymphoma (Figure 6). The patient completed a total of five cycles of R-EPOCH with complete remission documented by PET-CT at completion. The patient tolerated all chemotherapy cycles without significant complication or infection. A sixth cycle was considered but forgone due to the development of worsening vaginal-urethral fistula requiring ileal conduit creation, the complete response seen on PET-CT, and the desire to start treatment for her original diagnosis of vaginal carcinoma, which had already been delayed by lymphoma treatment.

The patient underwent surgery to create an ileal conduit with plans to start radiation therapy for her vaginal cancer after conduit creation. Surprisingly, during conduit surgery, a pelvic exam under anesthesia showed no residual vaginal tumor. Vaginal and vulvar biopsies performed at the time were also negative for squamous cell carcinoma.

Shortly after surgery, the patient experienced small bowel perforation. The patient underwent emergent laparotomy and bilateral salpingo-oophorectomy to investigate and treat the small bowel perforation. The pathologic diagnoses were diffuse CMV infection of the ovaries, fallopian tubes, small bowel, vagina, and urethra. A resected small bowel specimen also yielded histoplasma. The patient was initiated on anti-CMV and antifungal therapy but had a prolonged hospitalization due to repeated bloodstream and urinary tract bacterial infections and died from septic shock roughly three months after her initial ileal conduit creation.

## Discussion

The patient’s diagnostic journey, choice of lymphoma therapy, and ultimately fatal complications are illustrative of the complexities and precautions needed in the diagnosis and treatment of HIV-associated lymphoma and synchronous malignancies. DLBCL is the most common type of non-Hodgkin lymphoma in adults, including those with HIV infection [1]. The underlying pathobiology which increases the rate of lymphomas among HIV patients is incompletely understood. Various theories have been proposed. One maintains that lymphoproliferative neoplasms are more likely to develop due to the chronic antigen stimulation in patients who are immunodeficient and thus experience frequent clinical and sub-clinical infections, as emergence of a monoclonal B-cell population will eventually emerge from polyclonal B-cell stimulation. A second theory considers coinfecting oncogenic viruses, such as Epstein-Barr virus, that are more common in HIV patients. Another mechanism, not mutually exclusive with others, is immune deficiency leading to decreased immune surveillance of abnormal or malignant cell growth [2, 3].

HIV infection is a risk factor for a multitude of infectious and non-infectious, common and rare, and indolent and aggressive comorbidities, which makes the workup of a new complaint or new symptom more complex. In the above case, but for thorough workup, the patient may have been incorrectly diagnosed and treated for metastatic vaginal squamous carcinoma when in fact she had localized vaginal squamous carcinoma and DLBCL, both curable entities with vastly different treatment approaches that depend on accurate staging. Further, the diagnostic tools used in cancer diagnosis and staging have different positive predictive values depending on the patient population. Exemplified in this patient is the limited ability of PET imaging to stage a patient with untreated HIV, as what would usually be thought to represent metastatic manifestations of a primary tumor could also be synchronous malignancy, atypical opportunistic infection, or HIV itself. For this patient, this could have occurred not only during diagnosis but also during treatment restaging as inguinal lymph nodes initially thought to be residual lymphoma were in fact CMV adenitis. Emphasis should be placed on tissue biopsy, when practically feasible, to confirm any imaging finding whose etiology is in question.

Since the discovery of HIV and the advent of more refined antiretroviral treatment regimens, there has been debate on the impact HIV infection and its therapies have on lymphoma prognostication and treatment. A phase III study in 2005 by Kaplan et al. compared the use of the standard of care CHOP (cyclophosphamide, doxorubicin, vincristine, prednisone) regimen with and without the use of rituximab (R-CHOP) in HIV patients with high grade lymphomas. Unexpectedly, the rituximab containing regimen had increased rate of infectious deaths without a significant improvement in lymphoma outcomes. This study raised doubts regarding the use of rituximab in HIV-associated lymphomas, despite it having become the standard of care in non-HIV-associated lymphomas. However, the study’s conclusion was criticized by later sub-group analysis demonstrating that increased infectious risk was only in those with severely low CD4 T-cell counts [4]. A 2006 phase II study by Boue et al. employing R-CHOP for HIV-associated lymphoma did show complete remission and two-year overall survival rates of 77% and 75%, respectively, suggesting that rituximab is efficacious and safe in this clinical context [5].

Based on data in aggressive lymphoma patients suggesting that the more aggressive regimen of R-EPOCH may be more efficacious in inducing complete remissions, two trials investigated its use in the HIV infected population. Sparano et al. utilized various schedules of R-EPOCH and compared it to outcomes utilizing R-CHOP by retrospective analysis, concluding that R-EPOCH was superior [6]. Dunleavy et al. utilized a strategy of “short-course” R-EPOCH, treating patients with only one cycle past complete remission on PET. In that trial, 79% of patients only required three cycles, five-year progression free survival was 84%, and no treatment related deaths or new opportunistic infections occurred during chemotherapy [7]. This shorter duration of an intense chemotherapy is appealing in a population which is already immunodeficient and burdened by other comorbid conditions. A larger 2012 pooled analysis by Barta et al. concluded that R-EPOCH was superior to R-CHOP in HIV-associated non-Hodgkin lymphoma. Additionally, CD4 count <50/μL were associated with higher risk of treatment related death [8]. Based on these studies, R-EPOCH is the preferred National Comprehensive Cancer Network regimen for HIV-associated DLBCL, and the use of rituximab is definitively recommended only for patients with CD4 counts >50/μL [9].

The patient described above did indeed have a complete remission with an R-EPOCH regimen. Despite a CD4 count >50/μL even before starting on HIV therapy, she was not protected from opportunistic infection and death. While the CD4 count may have been protective in the cohort studied by Barta and Dunleavy, she was likely at higher risk due to her original vaginal cancer; she had no infectious complications during R-EPOCH but did succumb to infectious complications after ileal conduit operation necessitated by a large malignant vaginal-urethral fistula. 

One must therefore look beyond CD4 count as the sole predictor of infectious risk in HIV-associated lymphoma patients and consider other significant comorbidities such as synchronous malignancies. One strategy that may have addressed the added risk in this patient would have been to utilize the “short-course” R-EPOCH described by Dunleavy et al., as stopping after three cycles if permitted by PET may have prevented continued worsening of her fistula and given more time to heal prior to surgery. A shorter course of R-EPOCH would have also accelerated the time to begin definitive treatment for her vaginal carcinoma, which interestingly and unintentionally responded to R-EPOCH, with no evidence of disease during her subsequent exploratory surgeries and biopsies.

## Conclusions

This case highlights the complexities of diagnosing, treating, and monitoring patients with HIV-associated malignancies. Emphasis must be placed on a thorough workup, staging, confirmation of presumptions, and a heightened awareness of complications. HIV-associated lymphoma has been increasingly recognized as a lymphoma with unique attributes. This has led to increased study over the past decade in improving chemotherapy choices, optimizing treatment duration, and assessing the safety of high dose chemotherapy with autologous stem cell transplantation. This work holds promise in treating this challenging cohort and minimizing toxicity.
